# Rapidly Destructive Inflammatory Arthritis of the Hip

**DOI:** 10.1155/2014/160252

**Published:** 2014-07-07

**Authors:** Jenny Shu, Ian Ross, Bret Wehrli, Richard W. McCalden, Lillian Barra

**Affiliations:** ^1^Department of Medicine, Division of Rheumatology, Western University, London, ON, Canada N6A 5W9; ^2^Department of Diagnostic Radiology, Western University, London, ON, Canada N6A 5W9; ^3^Department of Pathology, Western University, London, ON, Canada N6A 5A5; ^4^Department of Orthopaedic Surgery, Western University, London, ON, Canada N6A 5A5; ^5^Division of Rheumatology, Arthritis Centre, Monsignor Roney Building, St. Joseph's Health Care London, 268 Grosvenor Street, London, ON, Canada N6A 4V2

## Abstract

Rapidly destructive coxarthrosis (RDC) is a rare syndrome that involves aggressive hip joint destruction within 6–12 months of symptom onset with no single diagnostic laboratory, pathological, or radiographic finding. We report an original case of RDC as an initial presentation of seronegative rheumatoid arthritis (RA) in a 57-year-old Caucasian woman presenting with 6 months of progressive right groin pain and no preceding trauma or chronic steroid use. Over 5 months, she was unable to ambulate and plain films showed complete resorption of the right femoral head and erosion of the acetabulum. There were inflammatory features seen on computed tomography (CT) and magnetic resonance imaging (MRI). She required a right total hip arthroplasty, but arthritis in other joints showed improvement with triple disease modifying antirheumatic drugs (DMARD) therapy and almost complete remission with the addition of adalimumab. We contrast our case of RDC as an initial presentation of RA to 8 RDC case reports of patients with established RA. Furthermore, this case highlights the importance of obtaining serial imaging to evaluate a patient with persistent hip symptoms and rapid functional deterioration.

## 1. Introduction

Rapidly destructive coxarthrosis (RDC) of the hip joint was initially coined in 1970 [1] to describe a rare syndrome that involved rapid and total deterioration of both the acetabular and femoral aspects of the hip joint [2]. The joint destruction is usually unilateral, occurs within 6–12 months of symptom onset, and has similar radiographic features of, but not consistent clinically with septic arthritis [3]. However, RDC is an idiopathic condition with no single diagnostic laboratory, pathological, or radiographic finding [4]. Several underlying pathogeneses proposed in the past include crystal deposition [5], neuropathic Charcot arthropathy [6], primary osteonecrosis [1], a subset of osteoarthritis [7], subchondral insufficiency fracture [8], and inflammatory arthritis [3, 9–11]. There have only been 8 case reports of patients with RDC ad preexisting rheumatoid arthritis (RA) (disease duration 2–20 years), some of whom were also treated with DMARDs and/or biologic therapy and required total hip arthroplasty [3, 9–11]. We report the first case of RDC as an initial presentation of seronegative RA.

## 2. Case Presentation

A 57-year-old Caucasian woman was referred to our rheumatology outpatient center from the orthopedics service for assessment of a possible inflammatory etiology for her rapidly destructive arthritis.  Table 1 summarizes the major clinical, lab, and imaging findings. She initially presented with 6 months of progressive right hip and groin pain with no preceding trauma or chronic steroid use. There was a leg length discrepancy with the right leg 3 cm shorter and severe limitation of the right hip and some decreased range of motion on internal and external rotation of her left hip. She did not have any neurovascular compromise. Over 5 months, she became severely disabled and was unable to ambulate. With respect to her other joints, she had chronic pain in her metatarsal phalangeal joints (MTPs) and toes for approximately 3 years with progressive deformities, with recent episodes of swelling. Subsequent to her right hip pain, she also developed right knee pain with multiple episodes of warmth and swelling. Morning stiffness in affected joints was approximately 2 hours. Her initial swollen joint count (SJC) was 8 out of 66 joints examined, involving her right knee and multiple metatarsal phalangeal joints (MTPs). Over the next few visits, her swollen joint count of her small joints reached 11. There was also an unintentional 50-pound (lb) weight loss since the onset of her illness, partially due to decreased appetite secondary to pain.

Initial diagnostic work-up was significant for elevated inflammatory markers, weakly positive antinuclear antibody (ANA) and otherwise negative autoimmune markers including both rheumatoid factor (RF) and anticitrullinated peptide (anti-CCP) (Table 1). All cultures, including three sets of anaerobic and aerobic cultures and one set of systemic fungal and mycobacterial culture, were negative. Metabolic panel showed normal renal, thyroid, and liver function. Angiotensin-converting enzyme (ACE) serum level was within normal limits. Malignancy work-up was negative: serum and urine electrophoresis, CEA, CA-125, total body position-emission tomography (PET) scan, and bone scan were all within normal range. Two incidental pulmonary nodules were found on computed tomography (CT) thorax with focal ground glass appearance, but negative for malignancy on bronchoscopy and on repeat imaging. CT abdomen and pelvis was negative for any abdominal masses and showed a soft tissue calcified mass in the right sacroiliac (SI) fossa and right gluteal muscles.

**Figure 1:**
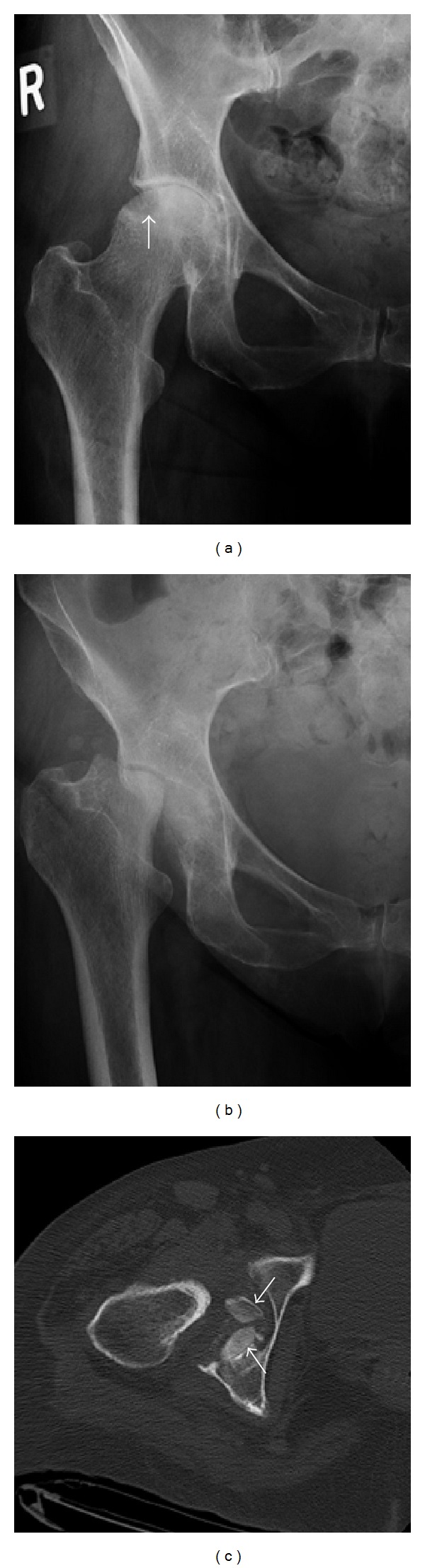
(a) AP radiograph of the right hip. A focal area of subchondral lucency is present involving the superolateral aspect of the right femoral head (arrow). (b) The follow-up radiograph taken 5 months later reveals near complete destruction of the femoral head. (c) CT scan of the right hip in the axial plane shows loss of the femoral head with two bone fragments within the hip joint (arrows).

**Figure 2:**
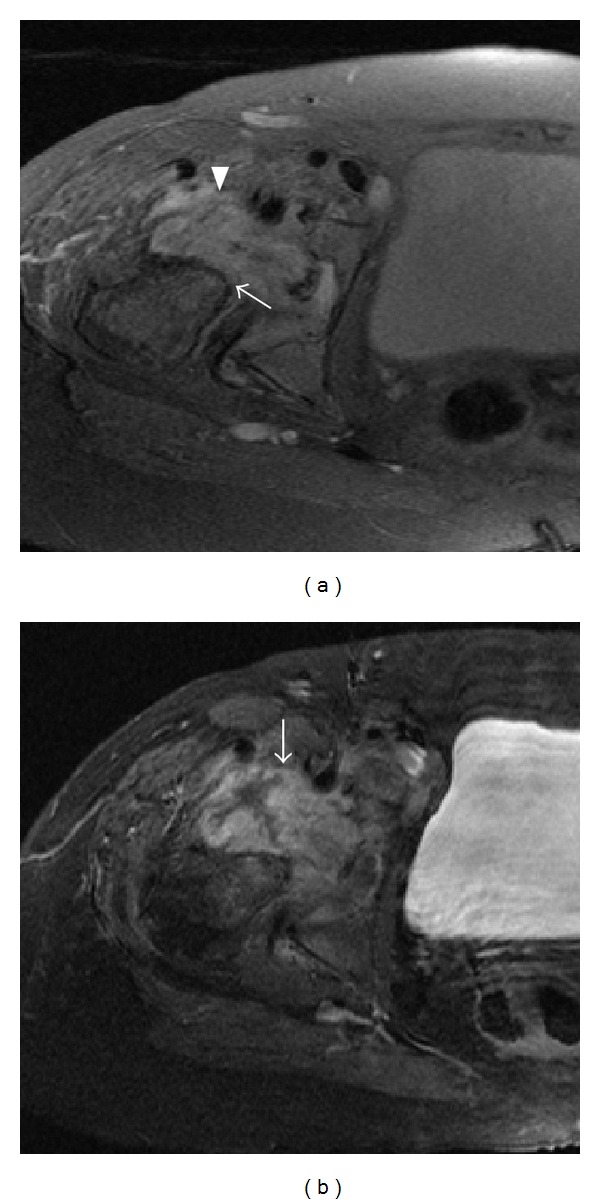
(a) Axial proton density with fat saturation sequence through the right hip joint (a) shows destruction of the femoral head (arrow) and a complex joint effusion (arrowhead). (b) Axial T1 fat saturated sequence after gadolinium reveals synovial thickening and enhancement (arrow).

Initial plain films of her right hip and pelvis showed femoral head lucencies compatible with subchondral cysts (but no definite fracture) and moderate diffuse articular joint space loss with flattening of the femoral head (Figure 1(a)). Over a 5-month span, there was complete destruction of the right femoral head, erosion of the right acetabulum, and lateral subluxation of the proximal femur (Figure 1(b)). CT pelvis with contrast (Figure 1(c)) showed fragmented bone within the acetabular fossa, which was remnants of the femoral head resorption process. Magnetic resonance imaging (MRI) of the right hip showed extensive synovial hypertrophy consistent with inflammatory arthritis (Figure 2). There were also minimal bone marrow edema and a fluid collection in the iliopsoas bursa extending posteriorly to the sciatic notch and enlargement of the hip joint capsule (Figure 2). X-rays of her feet revealed erosive changes in the MTPs and X-rays of her hands showed periarticular osteopenia in her metacarpal phalangeal (MCP) joints and ulnar deviation. There were degenerative changes on imaging of her knees, shoulders, and spine.

Three right hip aspirations were attempted with sufficient sample in only one attempt, which showed bloody fluid, 0.6 nucleated cells (17% neutrophils), and presence of only extracellular but not intracellular calcium pyrophosphate dehydrate (CPPD) crystals. Synovial biopsy did not reveal any crystals. Historically, CPPD crystal deposition disease can cause such acutely destructive disease on imaging and pathology [5], but the most common sites of CPPD joint involvement are the knees, wrists, and symphysis pubis with hip involvement being rarer with a prevalence of 5% [12]. This patient's plain-film images of her hands, knees, and pelvis were helpful in that there were no typical features of crystal arthropathy such as cartilage or joint capsule calcification and her blood work was also negative for an underlying metabolic precipitant of CPPD. Furthermore, single joint aspiration of her right knee showed no crystals, with bloody fluid and 35 nucleated cells (96% neutrophils).

The major differential diagnosis of this atypical case of destructive arthritis is outlined in Table 2. The patient's initial plain films showed evidence of degenerative changes but very unlikely to be primary osteoarthritis given the atypical symmetric joint space narrowing on plain imaging, complex joint effusion, and synovial thickening with chronic inflammatory changes on biopsy. She also did not have any evidence of a subchondral insufficiency fracture, which has been linked to the pathogenesis of the rapid destruction of osteoarthritic joints [8]. Other potential etiologies that could rarely cause such severe arthritis were considered on the differential including systemic diseases such as multicentric histiocytosis and sarcoidosis, but the patient lacked any other features of these diseases. Her neurovascular status was intact throughout and no evidence of a neurological problem or predisposing factors such as diabetes to cause Charcot's or neuropathic arthropathy. An avascular type necrosis (AVN) with subsequent inflammation was possible, but unusual without a history of steroidal use prior to her initial presentation or other risk factors for AVN. The imaging was also not classic for AVN and the patient did not have monoarthritis. Although seronegative, she did not have any inflammatory back pain, dactylitis, enthesitis, DIP involvement, inflammatory bowel disease, psoriasis, or other features of seronegative spondyloarthropathy. X-rays of her spine and CT pelvis did not show evidence of sacroiliitis.

**Figure 3:**
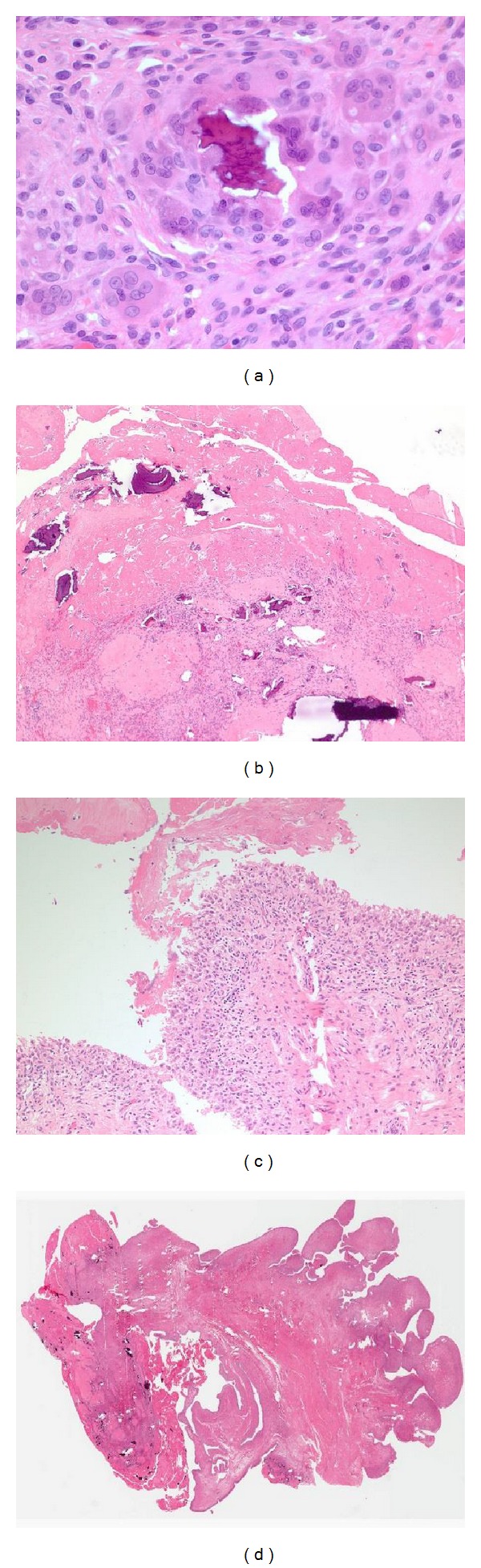
(a) Papillary, hyperplastic, chronically inflamed synovium is shown with abundant fibrin covering the surface and multiple fragments of bone being degraded by histiocytes and multinucleated giant cells. (b) At higher magnification, fibrin is seen on the surface of the synovium with a hyperplastic synovium consistent with chronic inflammation. (c) Fibrin and bone are detailed at 40x magnification showing multiple bone fragments which is typical of a rapidly destructive joint process. (d) Bone is seen being further broken down by multinucleated giant cells.

In order to definitively differentiate between chronic sepsis, malignancy, and a chronic inflammatory process, an open biopsy of the hip was performed by the orthopaedic service, which showed overall morphology with features of chronic inflammation, fibrosis, multinucleated giant cell reaction with dystrophic calcification, and reactive synovial proliferation (Figure 3). Cultures of synovial tissue were negative for fungus and mycobacteria, ruling out tuberculosis. Although the biopsy results were not specific for an exact etiology of rapid joint destruction, we were able to exclude neoplastic, infectious, osteoarthritis, and osteonecrotic etiologies.

An inflammatory etiology was most likely given multiple swollen joints, elevated inflammatory markers, constitutional symptoms, evidence of inflammatory features on imaging, and other causes excluded. Hence, a diagnosis of seronegative rheumatoid arthritis (RA) was ultimately made, fulfilling 4/7 of the 1987 RA American College of Rheumatology (ACR) classification criteria [13] and scoring 6 points for the 2010 ACR//European League Against Rheumatism (EULAR) classification criteria [14] (Table 3). Given the extent of right hip destruction, the patient received a total hip arthroplasty with good results. To prevent destruction of her other joints, triple disease modifying antirheumatic drugs (DMARD) therapy with hydroxychloroquine 400 mg daily, leflunomide 20 mg daily, and methotrexate 25 subcutaneously weekly was initiated. For symptom relief, she was given an 80 mg intramuscular Depo-Medrol injection and joint injection to her right knee. At her 2-month follow-up visit, she had significantly reduced swelling of her knees and MTP's with much symptomatic relief. Adalimumab was added because of incomplete response and patient had further improvement. Over a 6-month period, SJC decreased from 8 to 1/66.

## 3. Discussion

Inflammatory arthritis is a rare cause of RDC with only 8 case reports of patients with preexisting RA [3, 9–11]. Likewise, our case is the first with RDC as an initial presentation of RA. In contrast to this patient, seven of the cases were in patients with well-established rheumatoid arthritis (disease duration between 7 and 20 years) who all required total hip arthroplasties [3, 9, 10]. Another case [11] had a similar presentation with early RA of 2-year disease duration, but details on treatment were unavailable. All previously reported cases were of Japanese patients; whereas our patient is Caucasian. There was one case of a 45-year-old Japanese man with a 7-year history of RA refractory to betamethasone and methotrexate who developed RDC and an enlarged iliopsoas bursa and was subsequently treated with a biologic agent (etanercept) in addition to arthroplasty with good results [9]. Similarly, our patient had resolution of joint swelling in her knee and MTPs on subcutaneous weekly methotrexate, leflunomide, and hydroxychloroquine and adalimumab.

Another atypical feature of this patient's presentation was an associated benign soft tissue mass with enhancement involving the articular region, acetabulum, sacroiliac region, and anterior pelvis (Figure 2). A previous case report [11] has found evidence of iliopsoas bursitis in a patient with RA who developed rapidly destructive inflammatory hip arthritis with MRI findings similar to this patient who had a soft tissue mass that enhanced post gadolinium and was consistent with bursitis (Figure 2). Three other previous case reports also had nonmalignant inflammatory soft tissue masses on imaging [3, 9]. Iliopsoas bursitis has also been seen in other cases of RDC [8] and suggests evidence of an inflammatory process. A possible mechanism may be the overproduction of synovial fluid exerting intra-articular pressure weakening the capsule and allowing the protrusion of the synovial membrane of the hip joint into the iliopsoas bursa, which was seen with other cases of RDC of inflammatory etiology in patients with RA [11].

Total hip arthroplasties have been used as a treatment modality for severe RDC with good response in a retrospective review of total hip arthroplasties performed on patients with RDC [4]. However, thorough preoperative work-up should be completed to exclude contraindications to total hip arthroplasties particularly neoplasia, infection, and neuroarthropathy [4].

To conclude, rheumatoid arthritis is a rare cause of rapidly destructive coxarthrosis (RDC). We present the first case report of RDC as the initial presentation of seronegative rheumatoid arthritis in a 57-year-old woman who required right total hip arthroplasty, but whose other active joints had a good response to DMARD and biologic therapy. This case also highlights the importance of obtaining serial imaging to evaluate a patient with persistent hip symptoms and rapid functional deterioration.

## Figures and Tables

**Table 1:** Major clinical, lab, and imaging findings.

(1) Rapid progressive right hip destructive arthritis	
(2) Morning stiffness greater than 1 hour	
(3) Maximum active joint count of 11	
(4) Chronic metatarsal phalangeal joints arthritis with erosions, periarticular osteopenia on imaging of hands	
(5) Right knee arthritis with no imaging evidence of crystal arthropathy	
(6) Erythrocyte sedimentation rate (ESR) of 56 mm/h and C-reactive protein (CRP) 106.4 mg/L, antinuclear antibody (ANA) was weakly positive at 1:80, with a negative rheumatoid factor (RF), anticitrullinated peptide (anti-CCP), antidouble stranded DNA (anti-dsDNA), and extractable nuclear antigen (ENA) screen	
(7) Two incidental pulmonary nodules	
(8) A soft tissue calcified mass in the right sacroiliac (SI) fossa and right gluteal muscles	
(9) Presence of extracellular calcium pyrophosphate dehydrate (CPPD) crystals in right hip joint, negative synovial biopsy for crystals	
(10) Biopsy showed chronic inflammation, fibrosis, multinucleated giant cell reaction with dystrophic calcification, and reactive synovial proliferation	

**Table 2:** Major differential diagnosis of rapidly destructive coxarthrosis.

(1) Infectious particularly mycobacterial and fungal	
(2) Crystal arthropathy	
(3) Avascular necrosis	
(4) Inflammatory such as rheumatoid arthritis	
(5) Degenerative	
(6) Neuropathic	
(7) Seronegative spondyloarthropathy	
(8) Multicentric histiocytosis	
(9) Sarcoidosis	
(10) Neoplastic	

**Table:** (a) 1987 American College of Rheumatology (ACR)

Morning stiffness > 1 hour (1)	
Arthritis of 3 or more joint areas (1)	
Symmetric arthritis (1)	
Radiographic changes (1)	

Total: 4/7	

**Table:** (b) 2010 ACR/European League of Rheumatism (EULAR)

Greater than 10 small joints (4)	
Abnormal CRP and ESR (1)	
Symptoms > 6 weeks (1)	

Total: 6 points	

Table 3 details the clinical classification criteria of rheumatoid arthritis (RA) that the patient fulfills as part of the 1987 American College of Rheumatology criteria (A) and 2010 American College of Rheumatology/European League of Rheumatism criteria (B). Only the features the patient had that met criteria are shown with the number of points in brackets.
